# The interdependent complexity of disaster and Middle East Respiratory Syndrome

**DOI:** 10.4178/epih.e2016053

**Published:** 2016-11-28

**Authors:** Wonjae Lee

**Affiliations:** Graduate School of Culture Technology, Korea Advanced Institute of Science and Technology, Daejeon, Korea

## UNPREDICTABILITY AND COMPLEXITY OF RISK

The standard definition of risk and disaster can be likened to “being hit by lightning”. This has two different meanings. One is unpredictability because it does not occur through known mechanisms, and the other is that it is difficult to clearly analyze the events because cause and effect are statistically “independent” of each other (cf. International Organization for Standardization 31000).

However, there is a belief that today’s disease disasters such as Middle East Respiratory Syndrome (MERS) should be viewed as a complex development of “interdependent” events rather than as a chain of “independent” events. The initial development and spread of MERS have complicated connections to the response of the press and the government, which significantly decreases the possibility of control. This explains one of the reasons the models of medical-sociologists for disaster situations need to be based on “interdependence models” such as spatial analysis.

One of the general evaluations of MERS is “organized irresponsibility.” This is a paradoxical explanation that suggests collapse of organizational (systematic) responses based on responsibility. This is because disaster response organizations and entities have formed an impromptu and inefficient network in response to urgent situations.

The public response to disasters has high complexity due to interdependence. In the event of a disaster or a warning, the general public’s voluntary notification spreads through Social Networking Service and internet communication. While the propagation of disaster information in such private sectors is efficient in that it shows a speed that surpasses the existing alarm system, it can lead to secondary damage from the disaster by causing a paralysis of whole communication with freezing of communication lines, and providing the same propagation speed for false rumors.

## EMANCIPATORY CATASTROPHISM VS. DISASTER PREVENTION TRAINING

There are sociologists who concentrate on the liberating effects of disasters. They argue that new social solidarity is created from a collective reflection on existing institutions and technologies through disasters. However, analysis of our society’s reaction to MERS was more focused on the political gains and losses caused by the disaster instead of coming to an objective understanding of the incident. Thus, focusing on strengthening direct and technical training in response to disaster itself before expecting social understanding of the disaster will bring more practical and positive effects.

