# Supplementary material for: The interdependent complexity of disaster and Middle East Respiratory Syndrome
Source: Epidemiol Health. 2016 Nov 28;38:e2016053. doi: 10.4178/epih.e2016053 (PMC5177800; doi:10.4178/epih.e2016053)
Supplement: Supplementary file 1 [file epih-38-e2016053-supplementary.pdf]

재난의 상호의존적 복잡성과 메르스

- 이원재 (KAISTcf 문화기술대학원)

- 위험 (Risk) 의 예측불가능성과 복잡성

위험, 재난의 표준적인 정의는 “벼락에 맞은 것”에 비유할 수 있다 (ISO 31000). 여기엔 두 가지 의미가 있다. 하나는 알려진 기제를 통해 일어나는 것이 아니기 때문에 예측이 불가능하다는 것이고, 다른 하나는 원인과 결과가 통계적으로 “독립적”이기 때문에 일어난 사건을 명확하게 분석하기 어렵다는 점이다.

하지만 오늘날 메르스와 같은 질병 재난은 “독립적” 사건들의 연쇄보다는 "상호의존적" 사건의 복잡한 전개로 보아야 한다는 시각도 있다. 메르스의 최초 발생과 확산 과정은, 언론과 정부의 반응 등과 복잡하게 연결됨으로써 통제 가능성이 현격하게 떨어진다. 재난적 상황에 대한 의료-사회학자들의 모델이 공간 분석 (spatial analysis)와 같은 “상호의존 모델”에 기초해 고도화돼야 하는 이유가 여기에 있다.

세월호, 메르스에 대한 일반적 평가 중의 하나는 "조직화된 무책임함" 이다. 이는 역설적인 표현이다. 이는 실재로 책임의 소재를 기반으로 한 조직적 (organizational; systematic) 대응이 무너졌다는 점을 가리킨다. 이는 재난 대응 조직과 주체들이 질서 있는 태세가 아니라 급박한 상황 앞에서 즉흥적이고 비효율적인 네트웍을 형성했기 때문이다.

재난에 대한 대중의 반응 또한 상호의존성으로 인한 높은 복잡성을 갖고 있다. 재난의 발생과 경고의 경우 대중의 자발적인 신고가 SNS와 인터넷 통신을 매개로 퍼져나간다. 이 같은 사적영역의 재난 정보 전파는 한 편으론 기성 경보 시스템을 능가하는 속도를 보여준다는 점에서 효율적이지만, 다른 한 편으론 몇 군데 통신선의 마비만으로도 전체 통신이 마비될 수 있고, 잘못된 루머도 같은 정도의 전파 속도를 가진다는 점에서 재난의 2차 피해를 야기할 수 있다.

- 재난의 해방적 효과 (Emancipatory Catastrophism) vs. 대비 훈련

재난의 해방적 효과에 주목하는 사회학자들이 있다. 이들은 재난을 통해 기존의 제도와 기술에 대한 집단적 반성이 일어나고 이를 통해 새로운 사회적 연대가 생성된다는 것이다.

하지만 메르스에 대한 우리 사회의 반응을 분석해본 결과, 사건에 대한 객관적 이해로 나아가기 보다는 재난이 초래한 정치적 득실에 더 치중하는 것으로 나타났다. 따라서 재난에 대한 사회적 이해를 따지기 전에 재난 자체에 대한 직접적이고 기술적인 훈련을 강화하는 것이 더 실질적이고 긍정적인 효과를 가져올 것이다.
